# Accuracy on the preoperative assessment of patients with adolescent idiopathic scoliosis using biplanar low-dose stereoradiography: a comparison with computed tomography

**DOI:** 10.1186/s12891-020-03561-2

**Published:** 2020-08-18

**Authors:** Kwong Hang Yeung, Gene Chi Wai Man, Tsz Ping Lam, Bobby Kin Wah Ng, Jack Chun Yiu Cheng, Winnie Chiu Wing Chu

**Affiliations:** 1 Department of Imaging and Interventional Radiology, Faculty of Medicine, The Prince of Wales Hospital, The Chinese University of Hong Kong, Shatin, Hong Kong; 2 Department of Orthopaedics and Traumatology, Faculty of Medicine, The Prince of Wales Hospital, The Chinese University of Hong Kong, Shatin, Hong Kong

**Keywords:** Adolescent idiopathic scoliosis, Three-dimensional analysis, Intervertebral axial rotation, Intervertebral wedging, Kyphosis, Lordosis, Torsion, Biplanar radiographs, Computed tomography

## Abstract

**Background:**

Although computed tomography (CT) is commonly used to diagnose the scoliotic spine in patients with adolescent idiopathic scoliosis (AIS) preoperatively, it is limited by the high radiation and prone scanning position. Recently, a new biplanar stereoradiography (EOS) was used to image the scoliotic spine in an upright posture with significantly less radiation in non-severe AIS subjects. However, its reliability to assess preoperative AIS patients remains unreported. Hence, the purpose of this study is to compare the scoliotic curvature between prone (CT) and upright positions (EOS) in preoperative AIS patients.

**Methods:**

Thirty-three pre-operative AIS patients (mean age:18.4 ± 4.2) were recruited. EOS was used to scan the whole thoracic spine at upright position. Whereas on the same day, a conventional CT scan was used to evaluate the spine in prone position. The three-dimensional reconstruction of EOS and CT of the spine were then generated. Using previous validated techniques, multiple scoliotic parameters in both modalities were determined. The agreement between the two modalities was compared using the Bland-Altman test, whereas the correlation was assessed by the intraclass correlation coefficient (ICC).

**Results:**

The mean ICC (prone and upright) of intra-rater/inter-rater reliabilities for the measured parameters were 0.985,0.961/0.969,0.903, respectively. Thoracic Cobb angles, intervertebral wedging and lumbar lordosis correlated significantly between upright EOS imaging radiographs (62.9 ± 9.3°,6.4 ± 2.9° and 48.8 ± 12.4°) and prone CT (47.3 ± 10.0°,5.8 ± 2.7° and 27.9 ± 11.4°; *P* < 0.001). The apical vertebral wedging and apical intervertebral disc wedging showed a good correlation among the two modalities (upright, 6.5 ± 3.5° and 6.4 ± 2.9°; prone, 6.5 ± 3.6° and 5.8 ± 2.7°; R^2^ ≥ 0.94; *P* < 0.01). Similarly, there was significant correlation in apical intervertebral rotation (R^2^ = 0.834; *P* < 0.01) between the prone CT (3.4 ± 3.0°) and upright EOS (3.8 ± 3.2°). In addition, the Cobb angle was significantly larger in upright EOS (62.9 ± 9.3°) than in prone CT (47.3 ± 10.0°, *P* < 0.01) position. There was significant underestimation on scoliotic severity in the prone position when compared with upright position.

**Conclusions:**

Importantly, the image acquisition and reconstruction from EOS can better provide accurate three-dimensional spinal representations of the scoliotic curvature in preoperative AIS patients. Moreover, our findings suggested that scoliotic curvatures in preoperative AIS patients can be largely represented by both imaging modalities despite the difference in body positioning.

## Background

Adolescent Idiopathic Scoliosis (AIS) is a complex three-dimensional (3D) deformity of the spine with an unknown etiology [1]. Generally, it occurs mainly in adolescent girls during peri-pubertal age. Unlike a healthy individual with an erected spine, the spine of an AIS patient will grow a side-to-side curvature and appears as a sketched of “C” or “S” shape, depending on the number of curvatures. And most often, these curvatures are accompanied by rotation. Currently, patients with milder curvatures are treated by routine clinical monitoring and bracing to suppress the curve from further progressing. However, for those with a severe curve progression, surgical treatment would then be prescribed [2].

Prior to the spinal surgery being performed, imaging modalities would commonly be used to observe the scoliotic spine for surgical planning. Commonly, a computed tomographic (CT) scan will be used as a routine standard for this imaging assessment [3]. As in most institutions, a prone position in CT scanning will be used to mimic the position at surgery as closely as possible [4]. However, as CT is done in a non-standing position and have a relatively high radiation exposure, it may not be an optimal choice on adolescents with scoliosis [3]. Recently, studies have utilized a bi-planar low-dose stereoradiography (EOS) to capture the 3D parameters in spinal curvature [5–9]. This is done in an upright position to assess the scoliotic details on the frontal and lateral planes simultaneously [10]. Importantly, this imaging modality have a high reduction on radiation exposure than the common digital X-ray radiography [4]. Although previous studies have validated the accuracy between CT and EOS in non-severe AIS patients, there is still no current report on the validation using pre-operative AIS patients.

The objective of this prospective study was to evaluate the differences in 3D morphological spine parameters between CT and EOS imaging of the scoliotic spine of the AIS patients preoperatively. We hypothesized that the 3D reconstruction of EOS scan of the spinal vertebrae in severe AIS patients is comparable with the vertebral morphologic measurements of the patients’ CT scans and exposes the patients to lower medical radiation.

## Methods

### Study population

We prospectively recruited all consecutive patients with AIS who were needed surgical treatment in our institution between June 2015 and January 2017. Children with other spinal pathologies, such as early onset scoliosis, previous spinal surgery, neurological symptoms or neural axis abnormalities, syndromes associated with disorders of growth, or atypical left convex thoracic curves or right convex (thoraco) lumbar curves, were excluded. Patients were further included when they had undergone preoperative EOS and CT scans at our institution on the same day. Written informed consent was obtained from the subjects or the parents of minor subjects before participating in this study. Ethical approval was obtained from the ethics review board of the joint NTEC-CUHK clinical research ethics committee. All study procedures were conducted in accordance to the guidelines approved by the ethics committee and the Declaration of Helsinki.

### EOS imaging and 3D reconstruction

**Fig. 1:**
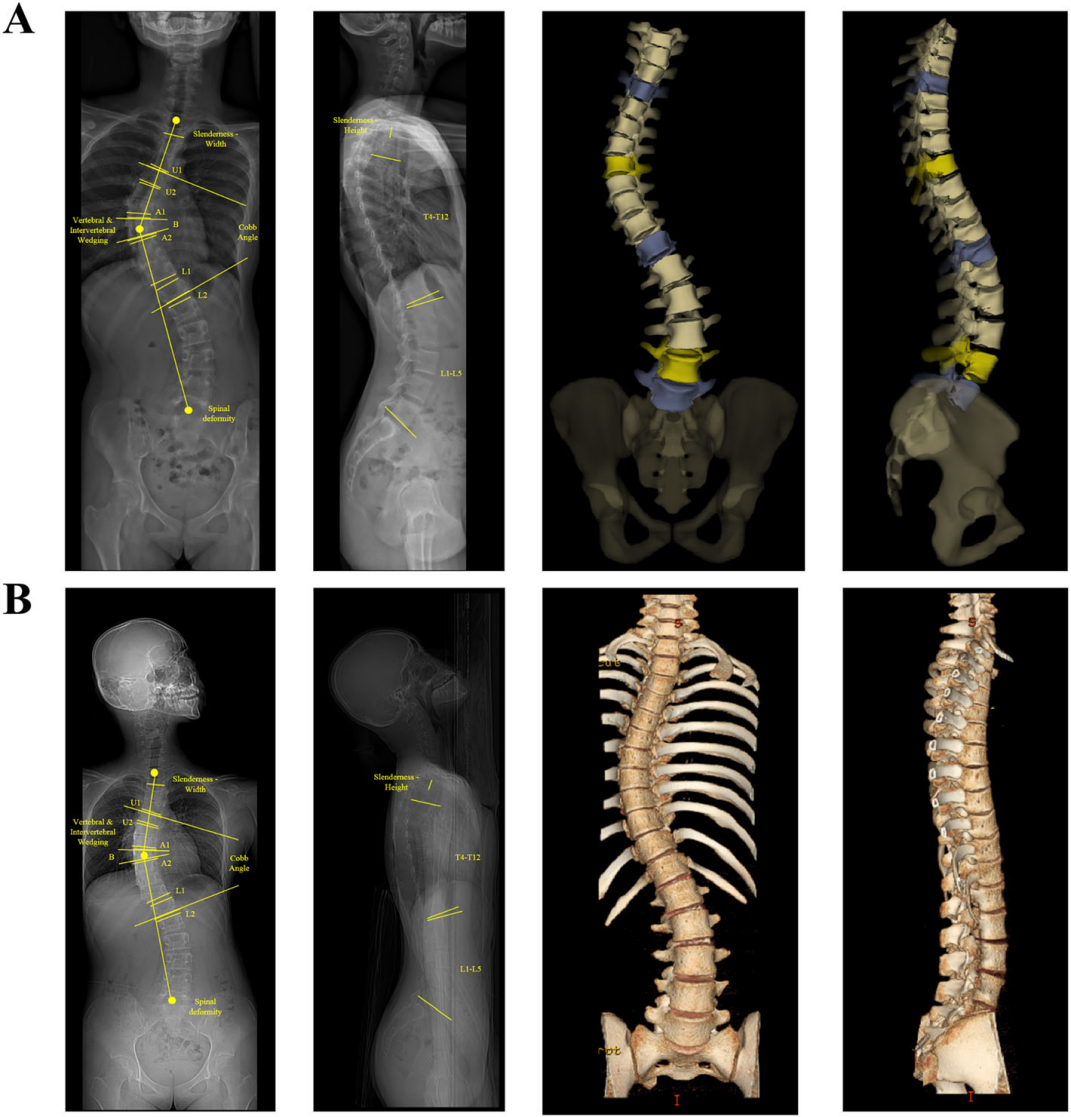
The radiographs of a 16-year-old female AIS patient with the frontal and lateral views, and 3D reconstruction images (**a** – EOS bi-planar stereoradiography; **b** – CT digital reconstructed radiography)

All subjects underwent whole body biplanar stereographs (EOS imaging, Paris, France) with a standardized radiographic protocol by a team of experienced radiographer. Subjects were instructed to stand in a comfortable position with hips and knees extended and with hands on a support. Upright EOS captured simultaneously the radiographs (Fig. 1a) of frontal and lateral views with two pairs of X-ray sources which positioned perpendicular to each other. The average scan speed used for EOS acquisition was 19.16 s. EOS images were then analyzed by the sterEOS workstation (Surgimap, Nemaris Inc., New York, NY) to generate the 3D surface reconstructions of each vertebra at each global axial rotation offset angle. Two trained observers, consisting of a senior consultant (B.K.W.N.) and an orthopaedics specialists (T.P.L.) with more than 30 years of experience in diagnosing AIS, reviewed the EOS scans. The measurements were performed twice for intra-rater reliability assessment. However, if there is an uncertainty between the two observers, a medical scientific officer (G.C.W.M.), with 14 years of experience in the field of scoliosis research, will helped with the diagnosis.

### CT imaging and 3D reconstruction

CT imaging (slice thickness of 0.625 mm, in-plane resolution of 0.352 mm/pixel, 64 Slice Multi-detector CT scanner, GE Healthcare, Chalfont, St. Giles, UK) was acquired in prone position, that was the standard workup in our medical centre during the inclusion period. The acquisition parameters for the CT imaging and 3D reconstruction is the following: 120 kV; 170 mA; rotation time = 0.8 s. The scan coverage in each case was from C7 to S1. Two trained observers, consisting of a Professor of Radiology (W.C.C.W) and a Professor of Orthopaedics and Traumatology (J.C.Y.C.) with more than 20 years of experience in diagnosing AIS, reviewed the CT scans. The measurements were performed twice for intra-rater reliability assessment. However, if there is an uncertainty between the two observers, a medical scientific officer (G.C.W.M.), with 14 years of experience in the field of scoliosis research, will helped with the diagnosis. In addition, a previously validated software and a semiautomatic image processing technique for CT scans of the scoliotic spine (ScoliosisAnalysis 4.1, Image Sciences Institute, Utrecht, The Netherlands, develop using MeVisLab, MeVis Medical Solutions AG, Bremen, Germany) were used to provide complete 3D coordinate systems of the individual structures of the spine [3]. By this method, the exact height of the osseous (anterior and posterior sides of the vertebral bodies, the laminae and the spinous processes) and non-osseous structures (anterior and posterior sides of the intervertebral discs, interlaminar spaces, and interspinous spaces) in the midsagittal plane were measured, while correcting for rotation and tilt in 3D (Fig. 1b). In contrast to the anatomical midsagittal plane of the patient, this complete 3D analysis method enabled the observer to reconstruct the midsagittal plane of each structure by taking account of axial rotation and coronal and sagittal tilt, as well as torsion (internal rotation) of each individual structure.

### Outcome measurements

**Fig. 2:**
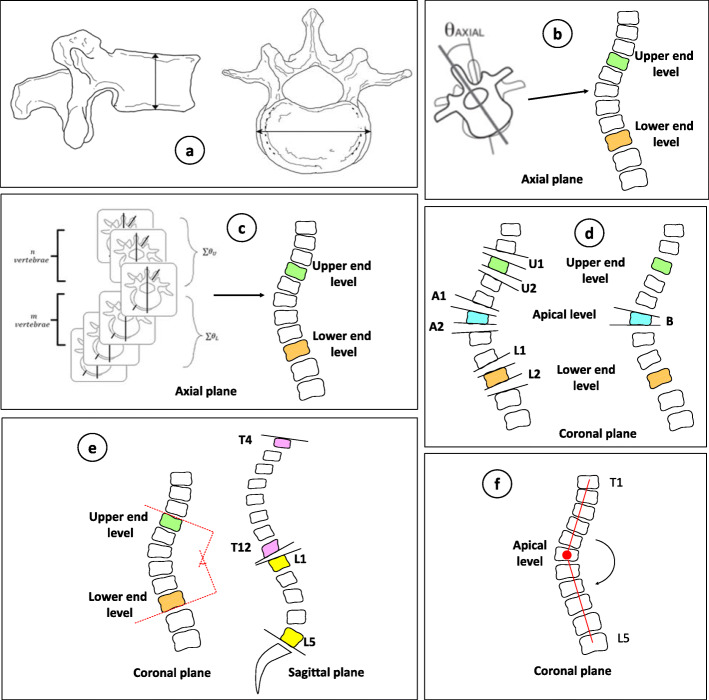
The calculated parameters were divided into 6 categories. (**a** – Slenderness (height/width ratio illustrated), **b** – Intervertebral axial rotation of the apex, upper, and lower junctional level and thoracolumbar level, **c** – Torsion, **d** – Vertebral and intervertebral wedging, **e** – Cobbs Angle, kyphosis and lordosis, and **f** – Spinal deformity)

All the EOS and CT images were collected from the Picture Archiving and Communications Systems (PACS) workstation (Carestream solution working station, Carestream Health, Version 11.0, Rochester, New York, USA). The calculated parameters were divided into 6 categories. Each category refers to global (whole spine), regional (scoliotic segment), and local (vertebra) descriptors. Vertebra centroid is understood as the halfway point between the centers of the 2 endplates of the vertebra. The local vertebra axis system is defined by the SRS 3D terminology group as follows (Fig. 2a – f):

1. Slenderness (The ratio of height over width) [11]

The height and the width were the distance in a coronal plane between superior and inferior endplates at the centre of the vertebra, and between left and right sides at the centre of vertebra which was perpendicular to the height line measured in a coronal plane from T1-L5 vertebrae, respectively.

2. Intervertebral axial rotation of the apex, upper, and lower junctional level and thoracolumbar level [12]

This is the measured rotation between two adjacent vertebrae in axial plane at apical, upper and lower curves, and thoracolumbar junction (T12-L1) vertebrae.

3. Torsion [13]

Mean of the sum of intervertebral axial rotation (measured according to the local referential of the inferior vertebrae) of the 2 hemicurvatures of the curve (between upper end vertebra and apex and between lower end vertebra and apex)

4. Vertebral and intervertebral wedging [11]

This is the measured angle between superior and inferior endplate of apical vertebra, between the disks and apical vertebra, between the disks and upper curve vertebra, and between the disks and lower curve vertebra.

5. Cobb angle [14]

Cobb angle is defined as the most tilted vertebrae above apex and below apex of a curve on coronal plane. Also, measured the angles on sagittal plane for thoracic kyphosis (T4-T12) and lumbar lordosis (L1-L5).

6. Spinal deformity

This was measured in the coronal plane from the angle between the centre of vertebra T1 to the centre of apex, and the centre of apex to the centre of vertebra L5.

All of the parameter results were compared between the prone (CT-generated DRR) and upright (EOS) positions.

### Statistical analysis

Using descriptive statistics to compute the means and standard deviations. A paired t-test was performed and compared the parameters between two scans. A Wilcoxon signed ranks test was used for the parameter with non-normal distributed data. The agreement between the two positions was tested according to the Bland-Altman plot; first, the one sample t test showed if there was a significant difference between the measurements; second, if there was no significant difference, the regression analysis showed if there was agreement between the measurements [15]. The correlations between two scans in the measurements were analyzed with a Pearson correlation test. The intra- and inter-observer reliability were obtained as intraclass correlation coefficients (ICC). *P*-value less than 0.05 was considered statistically significant for all analyses. All analyses were conducted with the SPSS software (Version 25.0; SPSS, Chicago, IL, USA).

## Results

### Patient demographic data

A total of 33 pre-operative AIS patients were recruited during the study period. This consisted of 26 females and 7 males with a mean age of 18.4 ± 4.2 years (range, 13–31 years) and mean Cobb angle of 62.9 ± 9.3°. There were 29 thoracic, 2 thoracolumbar and 2 lumbar curves. Most of the curves were classified as Lenke type 1 and 2 of the severe AIS patients. All descriptive statistics are shown in Table 1.

**Table 1:** Demographic data was presented for all the included AIS patients with EOS and CT scans

Demographic parameter	
No. of Subjects, n	33
Age at radiograph (years)	18.4 ± 4.2
Gender, n (%)	
Female	26 (78.8)
Male	7 (21.2)
Cobb angle (°)	62.9 ± 9.3
Type, n (%)	
RT	26 (78.8)
RT-LL	5 (15.2)
LL-RT	1 (3.0)
Triple	1 (3.0)
LTL	0 (0.0)
Other	0 (0.0)
Anthropometric data	
Height (cm)	159.8 ± 8.3
Weight (kg)	49.6 ± 8.0
BMI (kg/m^2^)	19.5 ± 3.0
Armspan (cm)	161.7 ± 9.8
BMI with Armspan (kg/m^2^)	19.0 ± 2.7

Data expressed as Mean ± Standard deviation; Data in bracket represent percentage

*n* sample size, *RT* right thoracic, *RT-LL* right thoracic-left lumbar, *LL-RT* left lumbar-right thoracic, *LTL* left thoracolumbar; Other, left thoracic, right lumbar, *BMI* body mass index

### Reliability of the measurements between CT and EOS imaging

The intraclass correlation coefficient (ICC) of intra-rater reliabilities for all scoliotic parameters at prone and upright positions were ≥ 0.913 and ≥ 0.878, respectively. The ICC of inter-rater reliabilities for all scoliotic parameters at prone and upright positions were ≥ 0.930 and ≥ 0.706, respectively. Overall, the ICC data suggested excellent measurement consistency and reliability in both the prone and upright positions (Table 2).

**Table 2:** Intraclass Correlation Coefficient (ICC) for the Intra-observer and Inter-Observer Reliability

	Intra-observer reliability		Inter-observer reliability	
Parameters	Prone CT	Upright EOS	Prone CT	Upright EOS
Slenderness	0.998 (0.308–1.000)	0.998 (0.105–1.000)	0.972 (0.806–1.000)	0.959 (0.862–1.000)
Cobb angle	0.996 (0.963–1.000)	0.993 (0.933–0.999)	0.979 (0.817–0.998)	0.985 (0.865–0.998)
Spinal deformity	1.000 (0.849–1.000)	0.986 (0.639–1.000)	0.973 (0.799–1.000)	0.871 (0.956–1.000)
Vertebral and intervertebral wedging	0.999 (0.993–1.000)	0.999 (0.994–1.000)	0.968 (0.850–0.994)	0.982 (0.912–0.996)
Kyphosis	0.913 (0.176–0.994)	0.878 (0.700–0.992)	0.930 (0.284–0.995)	0.706 (0.453–0.978)
Lordosis	0.991 (0.871–0.999)	0.911 (0.163–0.994)	0.981 (0.748–0.999)	0.888 (0.043–0.992)
Intervertebral axial rotation	0.996 (0.979–0.999)	0.959 (0.810–0.992)	0.978 (0.897–0.996)	0.933 (0.703–0.986)

ICC (95% Confidence interval)

### Comparison of the spinal parameters between at prone CT and upright EOS imaging

**Fig. 3:**
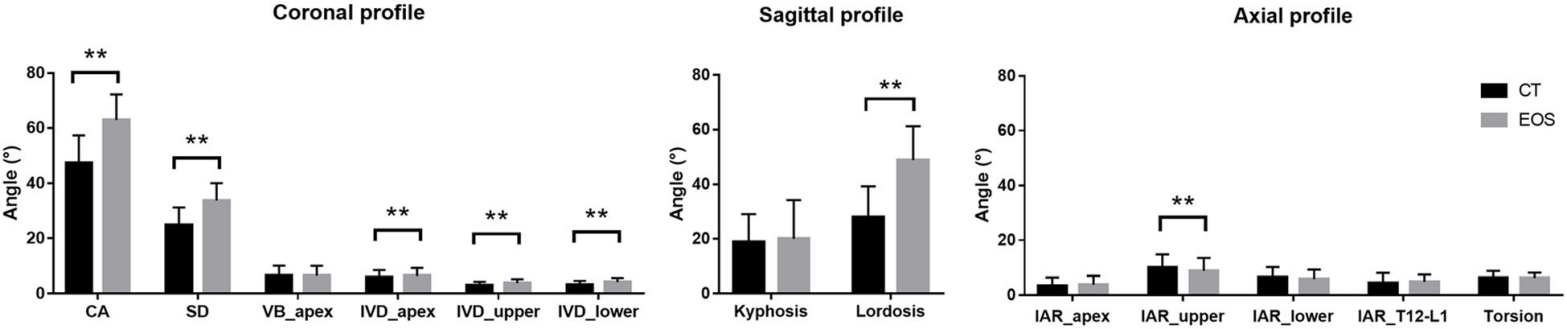
Mean values of all parameters measured in prone (CT) and upright (EOS) positions. CA, Cobb angle; SD, spinal deformity; VB, vertebral body; IVD, intervertebral disc; Apex, apical level; Upper, upper end level; Lower, lower end level; IAR, intervertebral axial rotation. Paired t-test: *P < 0.05 **P < 0.01

**Fig. 4:**
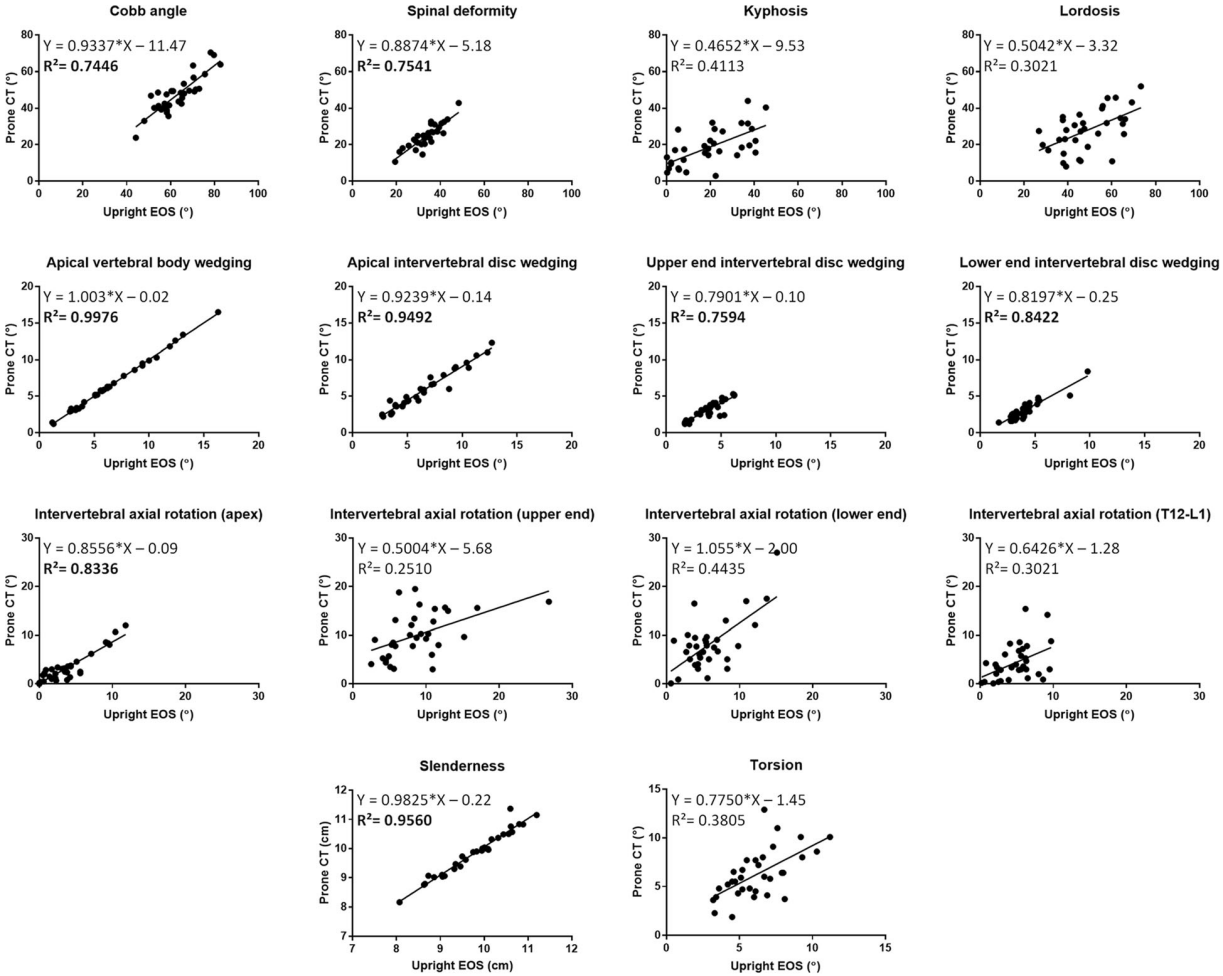
Scatterplot on the correlation of different scoliotic parameters between prone CT and upright EOS. Bold in **R**^**2**^ indicated the significance level: P < 0.01

Based on the coronal plane, no significant difference (*P* > 0.05) in slenderness was found between the prone CT and upright EOS (Table 3, Fig. 3). Likewise, the Cobb angle was significantly larger in upright EOS (62.9 ± 9.3°) than in prone CT (47.3 ± 10.0°, *P* < 0.01) positions as well as the spinal deformity (Table 3, Fig. 3). Although there was no observable difference in vertebral wedging being found, however, there was a significant difference in intervertebral wedging at apical, upper and lower end levels (*P* < 0.01) being found (Table 3, Fig. 3). And according to the Bland-Altman method, there was significant difference between Cobb angle, spinal deformity, vertebral wedging and intervertebral wedging in the prone (CT) and upright (EOS) positions (Table 3). In addition, all these parameters were significantly correlated between prone (CT) and upright (EOS) positions (R^2^ ≥ 0.75, *P* < 0.01; Fig. 4).

**Table 3:** Differences (mean ± standard deviation) between upright (EOS) and prone (CT) positions for different scoliotic parameters

Parameter		Position	Imaging modality	Mean ± SD	P-value
Slenderness (mm)		Prone	CT	9.74 ± 0.72	0.066 (0.898) ^*a*^
		Upright	EOS	9.69 ± 0.72	
**Coronal profile**					
Cobb angle (°)		Prone	CT	47.3 ± 10.0	< 0.001**
		Upright	EOS	62.9 ± 9.3	
Spinal deformity (°)		Prone	CT	24.8 ± 6.4	< 0.001**
		Upright	EOS	33.8 ± 6.3	
Vertebral wedging (°)	At apical level	Prone	CT	6.5 ± 3.6	0.921 (0.615) ^*a*^
		Upright	EOS	6.5 ± 3.5	
Intervertebral wedging (°)	At apical level	Prone	CT	5.8 ± 2.7	< 0.001**
		Upright	EOS	6.4 ± 2.9	
	At upper end level	Prone	CT	3.1 ± 1.2	< 0.001**
		Upright	EOS	3.9 ± 1.3	
	At lower end level	Prone	CT	3.2 ± 1.4	^ß^ < 0.001**
		Upright	EOS	4.2 ± 1.5	
**Sagittal profile**					
Kyphosis (°)		Prone	CT	18.8 ± 10.3	0.554 (0.024)
		Upright	EOS	20.0 ± 14.2	
Lordosis (°)		Prone	CT	27.9 ± 11.4	< 0.001**
		Upright	EOS	48.8 ± 12.4	
**Axial profile**					
Intervertebral axial rotation (°)	At apical level	Prone	CT	3.4 ± 3.0	0.057 (0.382) ^*a*^
		Upright	EOS	3.8 ± 3.2	
	At upper end level	Prone	CT	10.1 ± 4.7	0.134 (0.994) ^*a*^
		Upright	EOS	8.8 ± 4.7	
	At lower end level	Prone	CT	6.4 ± 3.8	0.002 (0.001)
		Upright	EOS	5.9 ± 4.5	
	At T12-L1 level	Prone	CT	4.4 ± 3.7	0.447 (0.024)
		Upright	EOS	4.9 ± 2.6	
Torsion (°)		Prone	CT	6.3 ± 2.5	0.878 (0.114) ^*a*^
		Upright	EOS	6.2 ± 2.0	

According to the Bland-Altman plot, the *P* value showed if there is agreement by using the t test. If this test showed no significant different (P > 0.05), a regression analysis was performed to see is if there is agreement, written in brackets; ^*a*^ *Agreement according to the Bland-Altman plot*

Paired t-test: * P < 0.05, ** P < 0.01; ^ß^ Wilcoxon sign ranks test:* P < 0.05, ** P < 0.01 for the non-parametric parameter; SD, standard deviation; T12-L1, Thoracolumbar level

On the sagittal plane, no significant difference in kyphosis was found between prone CT (20.0 ± 14.2°) and upright EOS (18.8 ± 10.3°) (Table 3, Fig. 3). On the other hand, the prone LL (27.9 ± 11.4°) was significantly lower than upright lordosis (48.8 ± 12.4°) (P < 0.01) (Table 3, Fig. 3). And from the Bland-Altman analysis, kyphosis was in agreement between the prone and upright positions (Table 3). However, lordosis was also found to be significantly lowered in prone (CT) position than when measured at upright (EOS) position (Table 3). No significant correlation was found in both kyphosis and lordosis between two positions (Fig. 4).

While for the axial profile, there was no significant difference in intervertebral axial rotation at apical, upper and lower end levels, and the thoracolumbar (T12-L1) level being observed between prone (CT) and upright (EOS) (Table 3, Fig. 3). Additionally, no significant difference was found on torsion (*P* > 0.05) when compared between prone (CT) and upright (EOS) (Table 3, Fig. 3). According to the Bland-Altman method, there was agreement between the intervertebral rotation at apical level and torsion in the prone and upright positions (Table 3). However, there was significant correlation in intervertebral rotation at the apical level (R^2^ = 0.834; *P* < 0.01) between the prone CT and upright EOS (Fig. 4).

## Discussion

Although CT scan is the most commonly used clinical standard to provide an accurate 3D reconstruction for bony measurement during surgical planning [3], the high radiation exposure can limit its use on pediatric and adolescent patients, especially for repeated exposures during preoperative and postoperative evaluations. Likewise, as often the CT utilize a prone scanning position to mimic surgical planning, this non-weight bearing position on the spine can cause a considerable alternation to the spinal curvatures [16]. Hence, in recent years, EOS imaging was developed to overcome the above issues [17–19]. Previous studies have shown EOS imaging can accurately provide 3D spinal representations of scoliotic spinal deformities in non-severe AIS patients, as compared with conventional CT [4]. However, its reliability and accuracy in severe AIS requiring surgical attention remains unclear. This aim of this study was to investigate the relationship between the prone CT and upright EOS in all three planes of the body to visualize the scoliotic spine of preoperative AIS patients.

In our study, we found good agreement between the prone and upright positions in axial, coronal and sagittal reconstruction between the two imaging modalities. The axial reconstruction by upright EOS imaging was consistent with those measured with conventional prone CT. Similar to previous study, Glaser et al. proved that image acquisition and reconstruction provided by EOS was well significantly accurate in 3D spinal deformities of position, orientation, vertebral shape by comparing with CT [5]. Several studies also evaluated the accuracy of vertebra and femurs reconstructions [11, 20–23] and the reliability of 3D models created by EOS imaging system [22, 23]. In our result, slenderness and vertebral wedging measurement provided good evidence for the reliability of reconstruction in EOS by comparing with CT [4, 5, 24]. Obviously, the length and the width of vertebrae were not affected by the body posture. In addition, no significant difference in intervertebral axial rotation and torsion was observed and hence, neither axial rotation or torsion were not so affected by the body posture.

However, it was observed an underestimation of the deformation of the spine in the prone position as compared to that in the upright position, which there is a significant lower values of Cobb angle, lordosis, intervertebral wedging, and spinal deformity from CT scanning being found. On the other hand, there was no difference being observed toward kyphosis. To give an illustration, scoliotic rotational curvatures were affected by the body posture excepted kyphosis, axial rotation, and torsion. With this in mind, due to flexibility of the severe scoliotic curve [25], the rigid rib cages at the thoracic region were restricted with this exception due to the restraining effect [26, 27]. Furthermore, a significant lower deformation of the curvature in CT due to the patient position variability and shown by the parameter of spinal deformity. EOS provided a more representative measurement in an upright position which has a critical variation in the spinal curvature. Herein, the effect of weight-bearing is therefore of paramount importance on the spinal curvature in AIS when compared with CT or MRI lying horizontally [4, 5, 24, 28, 29]. In addition, based on our previous publication, we demonstrated that entrance-skin dose from micro-dose EOS system was 5.9–27.0 times lower at various regions compared with standard digital radiography (DR). Similarly, patients with AIS received approximately 16–34 times lesser organ dose from micro-dose x-ray as compared with the standard DR [17]. Hence, EOS is compatible with CT to be used in clinical assessment with much less radiation exposure being applied to patients, especially with young subjects during puberty, on repeated exposure.

However, there are some limitations that should be addressed in the current study. The sample size was comparatively small with the preoperative AIS subjects though there was a significant linear correlation. In addition, our study recruited a heterogeneous population of preoperative AIS subjects (rather than homogenous thoracic curvature). Moreover, as the lying position would definitely create an alteration to the scoliotic curvatures, the parameters on the prone CT scans could not directly be compared to the upright EOS radiographs. However, as there are still no upright CT scans available in the open market, further comparison for EOS and CT in the same position of spinal deformity could not be accessed at this stage.

## Conclusions

Based on our current finding, there was no significant different in kyphosis, axial rotation and torsion between the two scans. This might be explained by the restricted flexibility of the severe scoliotic curve at thoracic levels by the rib cage. Importantly, our results indicated an under-estimation in the 3D deformity of the scoliotic spine by prone CT at the scoliotic parameters of Cobb angle, lordosis, intervertebral wedging, and spinal deformity. On the other hand, the 3D reconstruction from EOS imaging can provide an accurate and reliable measurements of the vertebral morphology in preoperative AIS patients. Importantly, the application of EOS imaging in clinical assessment toward the preoperative diagnosis for scoliotic surgery should be employed for enhancing patient’s care.

## Data Availability

The datasets used and/or analyzed during the current study are available from the corresponding author on reasonable request.

## Abbreviations

3D: Three-dimensional
AIS: Adolescent idiopathic scoliosis
CT: Computed tomography
EOS: Bi-planar low-dose stereoradiography
ICC: Intraclass correlation coefficient
